# Editorial: Spirituality and Mental Health: Exploring the Meanings of the Term “Spiritual”

**DOI:** 10.3389/fpsyg.2022.963708

**Published:** 2022-06-28

**Authors:** Marcelo Saad, Elaine Drysdale, Everton Maraldi

**Affiliations:** ^1^Teaching and Research Center Department, Albert Einstein Israelite Hospital, São Paulo, Brazil; ^2^Department of Psychiatry, University of British Columbia, Vancouver, BC, Canada; ^3^Department of Religious Studies, Pontifical Catholic University of São Paulo, São Paulo, Brazil

**Keywords:** spirituality, mental health, faith, laicism (secularism), religiosity

Scientific studies have demonstrated positive and causal associations between spiritual well-being and good physical and mental health parameters in recent decades. Spiritual well-being has been shown to modulate neurovegetative functions, mainly through stress reduction (Saad et al., 2019; Peres et al., 2020). Furthermore, biological and psychological changes have been documented in acts of prayer, forgiveness, gratitude, contemplation, sacred rituals, and blessings (Blažević, 2021). Spiritual coping, or the use of faith-related practices and conceptions to deal with threatening situations positively, may be a valuable resource for well-being. On the other hand, spiritual struggles can aggravate stress and adversely affect patients' health.

Dimensions of spirituality in health care and research can also encompass the empirical study of spiritual and paranormal experiences, usually referred to as anomalous experiences in the academic literature. For example, studies of altered consciousness associated with mediumistic and spontaneous spiritual awakening experiences have important implications for the intersection between physiology, consciousness, and spirituality (Maraldi, 2021; Tressoldi et al., 2022). Near-death experiences are also intriguing phenomena that defy purely materialistic explanations.

Therefore, an awareness of the patient's spiritual values and experiences is paramount in providing high-standard healthcare training and clinical practice. However, health professionals and researchers addressing spiritual issues have the challenge of dealing with an enormous spectrum of spirituality and religious beliefs. The task is even more challenging because most health care courses do not prepare graduates in this field. Furthermore, professionals might be confronted by prejudiced behaviors and attitudes of colleagues or managers.

In addition, the term “spiritual” remains an open, fluid concept without a single, universally accepted definition across health care and research. More recently, additional secular forms of spiritual experiences have arisen from rationalistic and humanistic analytic thinking. For example, “spiritual-but-not-religious” is an increasingly popular self-designated faith affiliation (Saad and de Medeiros, 2020).

This Research Topic explored the intersection of various “spiritual” conceptions with health care and their resulting clinical implications. The collected material may interest practitioners or scholars involved in health or behavioral sciences. This topic aims not to find a one-size-fits-all solution for the many terminological issues in the field of spirituality but to expand the discussion and help advance awareness of the relevance and importance of spiritual dimensions in patient care. Such awareness may impact academic understanding, clinical practice, institutional programs, and government policies. The selection of articles invites us to reflect on the many interesting possibilities in the interface between spirituality and mental health.

## Papers on Conceptual Definitions and Research Tools

Sena et al. investigated the definitions of spirituality in the healthcare field, identifying its main dimensions after a systematic review of scientific journals. The authors constructed a framework representing spirituality as a quantifiable construct, and this framework may serve to increase understanding of this topic's complexity. Braghetta et al. developed a new scale to evaluate spirituality and then assessed its reliability and validity. The “Attitudes Related to Spirituality Scale” (ARES) proved to be a reliable, valid, and stable one-dimensional instrument for use in the Portuguese-speaking population and could be studied for validity in other cultures and languages.

## Papers on Spirituality-Religiosity Related to Physical-Mental Health and Wellbeing

Rathakrishnan et al. studied the mediator role of spirituality in fear and mental health associated with the COVID-19 pandemic among Malay adults. The results demonstrated that individuals with a high level of spirituality were more likely to have a lower level of mental health…! Maulina et al. examined associations among affective state, somatosensory amplification, and spirituality in Japanese and Indonesian university students. Despite significant differences between the cultures in spiritual beliefs, spirituality was shown to play a role in somatic symptoms and positive affect in both cultures.

Leal et al. examined a frequently held perception in the psychological literature that hope for a miracle could have harmful consequences in health care. They used two examples to illustrate the relative absence of negative consequences in praying for a miracle and concluded that this practice could be recognized as openness to life, bolstering hope, and recognition of reality. In a subsequent paper of the same group, de Freitas et al. reviewed models better to understand the role of a belief in miracles and assess the impact of miracle belief on health parameters. The conceptual model presented provides a greater understanding of a patient's belief in a miracle, including its role in personal experiences and its impact on health care.

## Papers on Spirituality-Based Health Interventions

Mossbridge et al. studied time perspective as having good thoughts about the past, awareness of present constraints, and adaptive planning for a positive future. They created a scalable time-travel narrative tool, the web application “Time Machine,” to support people feeling connected to a wise and loving future version of themselves. Rabeyron proposed a clinical approach for counseling individuals who have experienced unusual spiritual experiences in a distressing context. The author presents a model of psychodynamic psychotherapy for anomalous experiences. Such a non-judgmental and open listening approach may replace this distressing experience by one that becomes integrated and transformative.

## Papers on Spiritual-Religious Issues in Healthcare Settings

Cone and Giske conducted a study using a self-assessment survey tool to examine the spiritual care competencies of mental health staff in Norway and understand the perspectives of mental health staff in Scandinavian culture. Although small, the study revealed a need for spiritual care educational materials explicitly targeted at those who work in mental health. Borragini-Abuchaim et al. studied the opinion of undergraduate medical students at a national medical school in Brazil. The authors concluded that including topics of a spiritual dimension would be feasible. Result suggests that the dimension of patient spirituality/religious faith has already become a recognized component of contemporary medical care—see also Borragini-Abuchaim et al. for a corrigendum on the original paper.

## Papers on Uncommon Religious-Spiritual Experiences

Büssing studied “wondering awe,” a perceptive aspect of spirituality relevant to religious and non-religious persons. High gratitude and awe scores were found more often in older subjects, those with the highest well-being, and those who meditate or pray frequently. “Wondering awe” was associated with many positive items of existential well-being. Corneille and Luke studied spontaneous spiritual awakenings (SSAs), the subjective experiences characterized by a sudden sense of contact, union, or experience of oneness with some ultimate reality, the universe, ‘God,' or the divine. Temporal lobe lability and trait absorption (the tendency to have attention absorbed in a task) predicted these experiences.

Spindola-Rodrigues et al. evaluated the cognitive functioning of 19 spirit mediums who practice trance mediumship in Brazil. Neuropsychological tests showed that their cognitive functioning scores were equal to or higher than the median scores of Brazilians. Less experienced mediums, on the other hand, evidenced executive dysfunction and increased common mental disorders. Houran and Laythe tested the validity and practical utility of the “Haunted People Syndrome” concept (individuals who recurrently report various “supernatural” encounters). Authors conclude that the studied family's ordeal involved mental boundaries in the face of stressful circumstances, with histrionic and catastrophizing reactions.

## Author Contributions

MS, ED, and EM acted as Guest Editors of the thematic Research Topic. After they wrote together this Editorial, based on the collected papers. All authors contributed to the article and approved the submitted version.

## Conflict of Interest

The authors declare that the research was conducted in the absence of any commercial or financial relationships that could be construed as a potential conflict of interest.

## Publisher's Note

All claims expressed in this article are solely those of the authors and do not necessarily represent those of their affiliated organizations, or those of the publisher, the editors and the reviewers. Any product that may be evaluated in this article, or claim that may be made by its manufacturer, is not guaranteed or endorsed by the publisher.
